# Formation and Alterations of the Potentially Harmful Maillard Reaction Products during the Production and Storage of Brown Fermented Milk

**DOI:** 10.3390/molecules24020272

**Published:** 2019-01-12

**Authors:** Zhonghui Han, Jianxin Gao, Xiaomin Wang, Wenxiang Wang, Jing Dong, Yan Zhang, Shuo Wang

**Affiliations:** 1 College of Food Science and Engineering, Qilu University of Technology, Jinan 250353, China; hanzhonghui3511@126.com; 2 Tianjin Key Laboratory of Food Science and Health, School of Medicine, Nankai University, Tianjin 300071, China; wangxiaomin@mail.tust.edu.cn; 3 State Key Laboratory of Food Nutrition and Safety, Tianjin University of Science and Technology, Tianjin 300457, China; gaojianxin001@126.com (J.G.); wenxianggd@163.com (W.W.); djanhui2010@126.com (J.D.)

**Keywords:** brown fermented milk, α-dicarbonyl, 5-(hydroxymethyl)-2-furfural, acrylamide, flavour component, formation

## Abstract

To improve the quality and safety of brown fermented milk (BFM), the formation and alterations of potentially harmful Maillard reaction products (MRPs), including 3-deoxyglucosone (3-DG), methylglyoxal (MGO), 5-(hydroxymethyl)-2-furfural (HMF), acrylamide and flavour components were investigated during the browning, fermentation and commercial storage. MRPs were shown to be produced mainly during the browning stage. The levels of different substances varied during the fermentation and commercial storage stage. The proportion and type of carboxylic acids in the flavour components significantly increased during the fermentation stage. Browning index of milk during the browning stage was shown to be positively associated with the 3-DG (Pearson’s r = 0.9632), MGO (Pearson’s r = 0.9915), HMF (Pearson’s r = 0.9772), and acrylamide (Pearson’s r = 0.7910) levels and the total percentage of the flavour components from four different categories (Pearson’s r = 0.7407). Changes in physicochemical properties of BFM during production not only contribute to predict the formation of potentially unhealthy MRPs, but also Lactobacillus species used for the fermentation should be carefully selected to improve the quality of this product.

## 1. Introduction

Brown fermented milk (BFM) is a fermented beverage made from the skimmed milk powder and reducing sugar, which is produced using Maillard browning and *Lactobacillus casei* fermentation [1]. BFM has both effects on human health and a unique colour and flavour, and is, therefore, popular with consumers in China, Japan, Southeast Asia, Europe and other regions [1]. *Lactobacillus casei* has been widely used in other dairy products such as cheese and ice cream [2,3]. Studies have shown that *Lactobacillus casei* have positive effect on consumer’s health, such as modulates immune function and improves constipation [4,5].

Colour and flavour of many food items, such as bread, coffee, chocolate and cakes, are based on the Maillard reaction during food manufacturing [6,7]. The browning process, which takes advantage of the Maillard reactions between the amino acids and reducing sugars in the reconstituted milk, is crucial for the formation of the BFM colour and flavour. However, the Maillard reaction has been reported to generate a variety of potentially unhealthy Maillard reaction products (MRPs), such as α-dicarbonyl compounds (α-DCs) [8,9], 5-(hydroxymethyl)-2-furfural (HMF) [10] and acrylamide [11,12]. Additionally, Lactobacillus fermentation produces various volatile flavour components, such as carbonyl compounds and organic acids in yogurt [13]. However, little is known about the potentially harmful MRPs, flavour constituents and colour components generated during the manufacturing and storage of BFM. Therefore, it is necessary to investigate the formation and changes of the MRPs during the processing of BMF.

α-DCs are highly reactive intermediates in the Maillard reaction, which can convert amino acid and protein modifications to advanced glycation end products (AGEs). AGEs have been shown to reduce the nutritional quality of foods and to be involved in the development of several diseases, such as neurodegenerative diseases, diabetes and others [14,15,16]. HMF is generated through the Amadori rearrangement in the Maillard reaction or by the direct dehydration of sugars, and this compound was demonstrated to have carcinogenic effects on animals and human cells [17,18]. Furthermore, acrylamide is a neurotoxic, genotoxic and potentially carcinogenic substance, generated in the Maillard reaction between asparagine and the reducing sugars during thermal processing [19].

Microorganisms may affect the MRPs during fermentation [20,21]. Methylglyoxal (MGO), glyoxal and diacetyl were reported to be produced during the Lactobacillus growth [22]. Lactobacillus-based fermentation leads to the reduction in acrylamide formation in mixed rye bread [23]. Additionally, yeasts were shown to reduce the formation of HMF and acrylamide in foods [20,24], and the relationships between different MRPs during the storage of commodities have been determined, including the conversion of 3-deoxyglucosone (3-DG) to HMF in honey or beer [9,25].

Therefore, the aim of this study was to investigate the formation and alterations of 3-DG, MGO, HMF, acrylamide and flavour components during the manufacturing and commercial storage of BFM, in order to help improve the quality and safety of BFM and to assist in developing a regulatory strategy for this food item.

## 2. Results and Discussion

### 2.1. Formation and Alterations of the MRPs in BFM

The formation of 3-DG, MGO, HMF and acrylamide during the browning, fermentation and storage of BFM is presented in Table 1. During the browning, both of 3-DG and MGO levels significantly increased with time, and 3-DG concentration reached a maximum of 82.13 ± 0.64 mg·L^−1^ at 180 min after the initiation of the reaction, showing that these compounds are produced during the BFM browning. Additionally, 3-DG content was approximately 20-fold higher compared with that of MGO, indicating that the α-DCs with C6 skeleton are formed at higher levels during browning. Our results are in agreement with the reports examining the browning of dairy products, biscuits, and other foods [26,27,28]. HMF levels increased from 0.16 ± 0.03 to 0.53 ± 0.02 mg·L^−1^ with time, which supports previous studies analysing biscuit baking [29]. However, we observed a decreased rate of acrylamide formation during the BFM browning. Previous studies demonstrated that acrylamide is rapidly formed during the processing at high temperatures accompanied by low moisture, such as frying or baking [30,31,32,33], which indicates that the relatively low temperatures and high moisture content may be primarily responsible for low acrylamide formation rates.

During fermentation, we observed a decrease in 3-DG concentration with time, which was shown to be significantly different. Similar results were reported by [34], who demonstrated that 3-DG does not generate browning colour products, such as melanoidins, but instead preferentially generates the cleavage products under acidic conditions. Therefore, 3-DG participates in complex reactions and its concentration decreased during BMF fermentation. However, MGO and HMF levels increase with the fermentation time, while that of acrylamide does not change significantly. MGO was reported to be formed during Lactobacillus fermentation [22], and it may be produced in the Maillard reaction between the reducing sugars and amino compounds during BFM fermentation [27,35,36]. HMF levels increase due the acidic conditions induced by Lactobacillus fermentation, which accelerate the conversion of lactose into glucose and galactose, and lead to the rapid formation of HMF through Maillard reactions [12]. An additional source of HMF during fermentation is the dehydration and cyclisation of 3-DG formed during the browning stage [34]. These results suggest that the Lactobacillus fermentation plays an important role in the alterations in potentially harmful MRPs during BFM processing.

During the commercial BFM storage (4 °C, 21 days), 3-DG levels increased during the first five days and decreased afterwards, which was comparable to its previously reported changes in a probiotic drink, but its levels in other dairy products were shown to be lower [8,27]. MGO contents increased gradually for 10 days, and remained at the same levels afterwards. Therefore, α-DC levels were shown to be unstable at the early stages of the commercial BFM storage, while stabilising later. No changes in the HMF levels were observed, which was consistent with the levels determined in the infant milk stored at 20 °C, but not in that stored at 37 °C, in which the HMF levels were shown to increase [37]. Therefore, low storage temperatures may inhibit the formation of HMF in BFM. Acrylamide contents in the stored BFM were below the LOQ, mainly due to its low levels formed during the browning and fermentation, and the dilution effect during the final blending, indicating that this compound does not represent an important health risk factor for the BFM use.

### 2.2. Formation and Alterations in the Flavour Components of BFM

**Figure 1:**
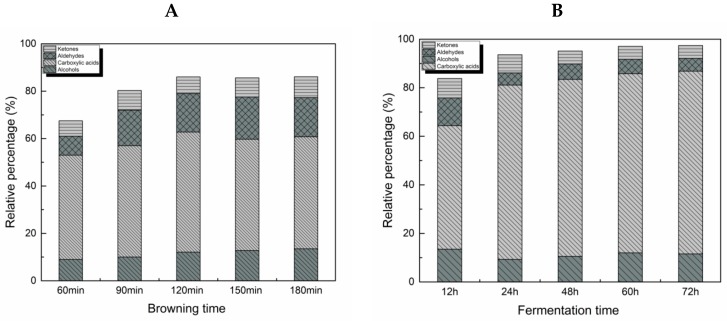
Changes in the relative percentage of flavour components identified in the brown fermented milk (BFM). (**A**) Changes during the browning stage. (**B**) Changes during the fermentation stage.

Total levels of flavour components were identified during the browning (180 min) and fermentation (72 h) of BFM. Browning stage flavours components mainly included carboxylic acids, followed by aldehydes, alcohols and ketones (Table 2). The proportion of these compounds in the total flavour components gradually increased with the browning time (Figure 1A). Flavour components such as acetaldehyde, 2H-pyran-2-one, 2-furanmethanol and furfural are produced during the browning stage and they provide the characteristic flavour of a product [6,38].

The number of flavour components identified in the fermentation stage was higher than that in the browning stage, of which the carboxylic acid species were particularly numerous, such as acetic and butanoic acids. The total proportion of carboxylic acids, aldehydes, alcohols, and ketones gradually increased during fermentation (Figure 1B). These results suggest that the carboxylic acids, such as acetic acid and butanoic acid, represent the most important flavour components in BFM, which was previously demonstrated in a study analysing fermented yogurt [13]. Moreover, the characteristic flavour components formed through the Maillard reactions were shown to exist during the fermentation process as well.

### 2.3. BFM Colour Alterations

The values of the colour parameters obtained during BFM browning and fermentation are presented in Table 3 (a*, b*, L*, ΔE* and BI). During browning stage, a* value increased from −2.16 ± 0.15 to 4.19 ± 0.06, while b* increased from 10.32 ± 0.08 to 16.65 ± 0.05 with the browning time, indicating that reconstituted milk lost greenness and blueness and became more red and yellow, respectively, during browning. The L* value, indicating the lightness of the sample, decreased from 88.79 ± 0.2 to 80.41 ± 0.04 with the browning time, indicating that the samples became darker in colour. Due to these results, the overall ΔE* values and BIs increased as well. The browning of the milk is primarily due to the Maillard browning of lysine and reducing sugars in the browned milk [39]. No significant changes in the colour of BFM were observed during the fermentation stage and commercial storage, most likely because of the lack of formation of coloured compounds.

### 2.4. Relationships between BIs, MRP Levels and Flavour Component Levels during the Browning of BFM

**Figure 2:**
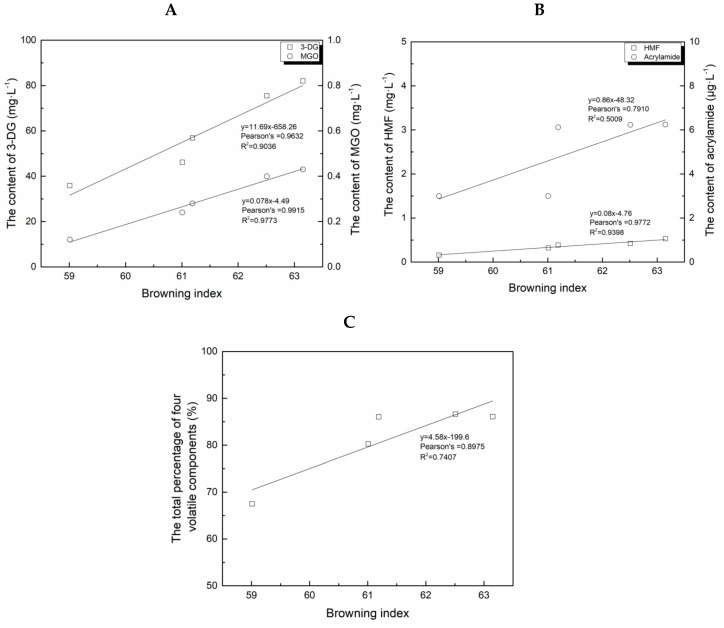
Correlations between the browning index (BI) and the levels of different compounds during the browning of brown fermented milk (BFM). (**A**) Correlations between deoxyglucosone (3-DG) and methylglyoxal (MGO) and BI changes. (**B**) Correlations between 5-(hydroxymethyl)-2-furfural (HMF) and acrylamide levels in BI changes. (**C**) Correlation between the changes in the total percentages of volatile compounds and BI alterations (*n* = 5).

Colour is an important indicator of browning, and it is usually used to determine the finalisation of the browning process. Maillard reactions are directly involved in the browning of BFM. BIs, MRP and flavour component proportions change with the temperature during Maillard reaction, as shown in Figure 2A–C.

Positive correlations between the BIs and 3-DG levels (Pearson’s r = 0.9632), MGO (Pearson’s r = 0.9915), HMF (Pearson’s r = 0.9772) and acrylamide levels (Pearson’s r = 0.7910) were observed, together with that between the BIs and the total percentage of flavour components from four described categories (Pearson’s r = 0.7407), indicating a strong correlation (|r| > 0.95) between the BIs and 3-DG, MGO and HMF levels during the browning of BFM. However, the correlations observed between the BIs and acrylamide and flavour components were weaker, indicating that the degree of browning can directly reflect the levels of potentially harmful MRPs during BFM browning. This may help improve the monitoring of MRP formation during the BFM manufacturing. Previous studies support the conclusion that the MRP levels correlate with the degree of browning in foods [6,40]. However, no correlation has been observed between MRP levels and BI changes during fermentation and storage of BFM, further confirming that the colour formed during the browning does not change during fermentation and storage.

The results of these MRPs, flavour and colour changes during BFM manufacturing and commercial storage provide a fundamental basis for improving the quality and safety of BFM, ensuring a stable, sustainable and healthy development of the dairy industry.

## 3. Materials and Methods

### 3.1. Materials

MGO, acetic acid (HPLC grade) and HMF were purchased from Sigma-Aldrich (St. Louis, MO, USA). Acrylamide and 3-DG were supplied by TRC (Toronto, Canada). d_3_-Acrylamide was supplied by Cambridge Isotope Labs (Cambridge, MA, USA), while o-phenylenediamine (OPD) was obtained from J & K Scientific (Beijing, China). Potassium hexacyanoferrate and zinc acetate were supplied by Sinopharm (Beijing, China). Water (HPLC grade) was prepared using a Milli-Q Integral purification system (Molsheim, France). Acetonitrile (HPLC grade) was supplied by Merck (Darmstadt, Germany). Skimmed milk powder (Fonterra, Taranaki, New Zealand) was purchased from the local supermarket. *Lactobacillus casei* 01 was supplied by Chr. Hansen (Beijing, China). Oasis HLB solid phase extraction (SPE) cartridges (6 mL, 200 mg) were supplied by Waters (Milford, MA, USA).

### 3.2. BFM Preparation

#### 3.2.1. Browning Stage

Skimmed milk powder was reconstituted to skim milk samples (120 g·kg^−1^) in water at 40 °C, and then glucose (40 g·kg^−1^) was added. The mixture was gently stirred for 30 min and heated to 65 °C. The sample was mixed homogenously at 20 MPa and browned at 95 °C for 180 min, which was followed by decreasing the temperature to 37 °C with cold water.

#### 3.2.2. Fermentation Stage

The browned sample was inoculated with the *L. casei* 01 strain at 2.5 × 10^6^ CFU mL^−1^, and incubated at 37 °C until titration acidity reached more than 200 (approximately 72 h). Afterward, the fermented curd was broken and cooled rapidly to 4 °C. Finally, the fermented milk, syrup and flavouring were mixed and homogenised at 25 MPa and stored in a refrigerator at 4–7 °C.

BFM preparation trials were performed in triplicate, and the mean values were reported.

#### 3.2.3. Quantification of 3-DG and MGO

Preparation of 3-DG and MGO was performed as previously described [41], with minor modifications. Briefly, 1 mL of sample and 50 μL of OPD (60 mg·mL^−1^) were added to a capped brown bottle. The mixture was incubated in water at 60 °C and stirred at 50 rpm. Following a 30-min incubation, the mixture was purified and concentrated using preconditioned Oasis HLB SPE cartridges. The samples were centrifuged at 10,000× *g* for 5 min and filtered through 0.22 μm nylon for LC/MS/MS analysis.

The derivatised and purified samples were analysed using an Agilent 1200 Series HPLC system coupled with Agilent 6410 triple-quadrupole MS (Agilent, Waldbronn, Germany) with electrospray ion source in positive ion mode and MRM mode. Nitrogen was used as the nebuliser (pressure, 40 psi), drying (flow rate, 10 L min^−1^ at 350 °C) and collision gas. The capillary voltage was 4 kV, while the dwell time was 80 ms for each transition. Chromatographic separations were performed on a Polar-RP 80A column (2 × 150 mm, 4 μm) maintained at 25 °C. A flow rate of 0.3 mL min^−1^ was used. The mobile phase consisted of phase A (0.1% formic acid aqueous solution) and phase B (methanol). The gradient was 20–80% B from 0–15 min and 80–20% B from 15–30 min. The sample injection volume was 5 μL. Transitions: 3-DG: *m*/*z* 199.1→*m*/*z* 145.1 and MGO: *m*/*z* 145.1→*m*/*z* 77. LOQs of 3-DG and MGO in samples were 11.5 μg L^−1^ and 1.5 μg L^−1^, respectively, while the LODs of 3-DG and MGO were 3.5 μg L^−1^ and 0.5 μg L^−1^, respectively. The recovery of these compounds was 86.6–92.7% and 84.8–90.2%, respectively. RSDs were 1.3–2.0% and 2.1–4.7%, respectively.

#### 3.2.4. Quantification of HMF

Samples (3 mL) were mixed with water (6 mL) and treated with 0.5 mL Carrez I and II solutions, each. The mixtures were vortexed for 3 min and then centrifuged at 4 °C for 15 min at 6000× *g*, to obtain clear supernatants. HMF was purified from the supernatants by using the preconditioned Oasis HLB SPE cartridges. The extracts were centrifuged at 10,000× *g* for 5 min, filtered (0.22 μm) and analysed with HPLC.

HMF was determined as previously described [42], with some modifications. Chromatographic analyses were performed using a Shimadzu HPLC system (Shimadzu, Kyoto, Japan) consisting of a CBM-20A system controller, LC-20A UV detector, two pumps (LC-20AT), a degasser (DGU-20A) and a column oven (CTO-20AT). Chromatographic separation was performed using HYPERSIL ODS-2 C18 analysis column (4.6 mm × 250 mm, 5 μm) at 35 °C. The flow rate was 0.6 mL min^−1^, and acetonitrile:water mixture (5:95, *v*/*v*) was used as a mobile phase for 20 min. The injection volume was 20 μL, and the UV detection wavelength was 284 nm. LOD and LOQ of the samples were 0.04 mg·L^−1^ and 0.12 mg·L^−1^, respectively. Recovery rates and RSDs were 80.2–85.7% and 4.0–5.5%, respectively.

#### 3.2.5. Acrylamide Quantification

Acrylamide was prepared as previously described [24], with minor modifications. Briefly, samples (3 mL) were mixed with water (6 mL) and treated with 0.5 mL of Carrez I and II solutions each. The mixtures were vortexed for 3 min and centrifuged at 5000× *g* for 5 min to obtain the clear supernatants. Acrylamide was purified from the supernatants using the preconditioned Oasis HLB SPE cartridges. Supernatants were centrifuged at 10,000× *g* for 5 min, filtered through a 0.22-μm nylon filter and analysed using LC/MS/MS.

Acrylamide quantitative analysis was conducted according to the method developed previously [12,43]. Samples were analysed using Agilent 1200 Series HPLC system coupled to the Agilent 6410 triple-quadrupole MS (Agilent, Waldbronn, Germany) with electrospray ion source in positive ion mode and MRM mode. Nitrogen was used as described for 3-DG and MGO, while the capillary voltage was 3.5 kV and the dwell time was 80 ms for each transition. Acrylamide separation was performed on Agilent ZORBAX Eclipse on C-18 column (2.1 × 150 mm, 3.5 μm). The mobile phase was methanol:water mixture (5:95, *v*/*v*), used for 15 min, and the flow rate was 0.2 mL min^−1^. The injection volume was 2 μL. Transitions used were acrylamide: *m*/*z* 72→*m*/*z* 55 and *d*_3_-acrylamide: *m*/*z* 75→*m*/*z* 58. LOD in samples was 3.0 μg L^−1^ and LOQ was 9.1 μg L^−1^. Recovery rates and RSDs were 83.8–89.7% and 1.9–4.5%, respectively.

#### 3.2.6. Colour Analysis

Colour measurements were obtained using Konica Minolta Spectrophotometer CM-5 colorimeter (Tokyo, Japan). The measurements were performed using a cuvette, colour parameters were CIE-L* (lightness), a* (greenness/redness), b* (blueness/yellowness) and ΔE* (total colour difference). Each sample was analysed six times. The *Δ*E* and BI (browning index) were calculated using the following equations [44].

(1) $$\Delta E^{*}=\sqrt{\left(\Delta L^{*2}+\Delta a^{*2}+\Delta b^{*2}\right)}$$

(2) $$\mathrm{BI}=100\left[\frac{0.31(a^{*}+1.75L^{*})}{5.645L^{*}+a^{*}-3.012b^{*}}\right]/0.172$$

#### 3.2.7. Flavour Components Analysis

Eight millilitres of samples were placed in a 50 mL vial. Volatile compounds were extracted by headspace solid-phase microextraction (HS-SPME) for 30 min at 55 °C using a DVD/Car/PDMS fibre (Supelco, Bornem, Belgium) with a multipurpose sampler. This method has been widely used to extract volatile and semivolatile components from dairy products [45]. Then, samples were directly desorbed into the injection port of the instrument which was at 250 °C. Gas chromatography–mass spectrometry (GC–MS) analysis of the SPME extract was performed using Varian 4000 (Varian, Walnut Creek, CA, USA) instrument equipped with Agilent DB-WAX capillary column (30 m × 0.25 mm × 0.25 μm). Carrier gas (helium) was set at a constant flow of 1 mL min^−1^, transfer line temperature was 280 °C and the ionisation voltage was 70 eV. A full-scan mode was run from *m*/*z* 30 to 500. Oven temperature was 40 °C for 3 min, followed by an increase to 150 °C at the rate of 3 °C·min^−1^, maintained at 150 °C for 2 min, increased to 250 °C and then maintained at 250 °C for 5 min. Mass spectra of unknown compounds were identified using the NIST MS Search 2.0 database. The percentages of compounds were calculated by the area normalisation method without considering, response factors.

#### 3.2.8. Statistical Analysis

Data were analysed using the analysis of variance (ANOVA) and Duncan test. Statistical analyses were performed using IBM SPSS 19.0 for Windows (SPSS Inc., Chicago, IL, USA). Correlations between the results were performed using OriginPro 2016 (OriginLab, Northampton, MA, USA). The values were considered statistically significant at *p* < 0.05.

## 4. Conclusions

Here, for the first time, the changes in the levels of potentially harmful MRPs, flavour components and colour were monitored during the manufacturing and storage of BFM. During BFM browning, MRPs (3-DG, MGO, HMF and acrylamide) were shown to be generated through the Maillard reaction and their levels significantly increase with the browning time. The changes in the MRP levels were demonstrated to correlate with the changes in the BIs, showing that these indices reflect the accumulation of potentially harmful MRPs in BFM and can be used for the monitoring of their levels, in order to improve the quality and safety of BFM. During fermentation, 3-DG levels in BFM decreased significantly, while the MGO and HMF levels increased; however, the levels of the MRPs did not significantly change during the commercial storage of BFM. Although alcohols, carboxylic acids, ketones and aldehydes are formed during the browning and fermentation stages, the most abundant components—carboxylic acids—are formed during fermentation. These results indicate that Lactobacillus fermentation plays an important role in the alterations of the MRP levels and flavours during BFM processing and storage, which depends on the metabolism of Lactobacillus species used for the production. The production of α-DCs was shown to be affected by Lactobacillus strains [22], and the selection and application of Lactobacillus strains that produce low levels of α-DCs and high levels of improved flavours during fermentation may represent a promising approach to the improvement of BFM quality.

## Figures and Tables

**Table 1:** Changes in the Maillard reaction product (MRP) levels during the manufacturing and storage of brown fermented milk (BFM).

**Browning Stage**	**Time (min)**	**3-DG (mg·L^−1^)**	**MGO (mg·L^−1^)**	**HMF (mg·L^−1^)**	**Acrylamide (μg·L^−1^)**
	60	35.90 ± 0.97 ^a^	0.12 ± 0.04 ^a^	0.16 ± 0.03 ^a^	-
	90	46.19 ± 1.11 ^b^	0.24 ± 0.02 ^b^	0.32 ± 0.02 ^b^	-
	120	56.91 ± 1.62 ^c^	0.32 ± 0.02 ^c^	0.38 ± 0.03 ^b c^	-
	150	75.57 ± 1.41 ^d^	0.40 ± 0.03 ^d^	0.42 ± 0.04 ^c^	9.32 ± 0.10 ^a^
	180	82.04 ± 0.64 ^e^	0.46 ± 0.01 ^d^	0.53 ± 0.03 ^d^	9.45 ± 0.12 ^b^
**Fermentation Stage**	**Time (h)**	**3-DG (mg·L^−1^)**	**MGO (mg·L^−1^)**	**HMF (mg·L^−1^)**	**Acrylamide (μg·L^−1^)**
	12	55.06 ± 1.20 ^a^	0.48 ± 0.01 ^a^	0.55 ± 0.02 ^a^	9.63 ± 0.10 ^NS^
	24	43.23 ± 0.52 ^b^	0.58 ± 0.04 ^b^	0.60 ± 0.03 ^a b^	9.56 ± 0.14
	36	35.92 ± 0.79 ^c^	0.81 ± 0.04 ^c^	0.62 ± 0.01 ^b c^	9.61 ± 0.18
	48	33.04 ± 0.56 ^d^	0.86 ± 0.09 ^c d^	0.67 ± 0.03 ^c d^	9.49 ± 0.19
	60	16.12 ± 1.40 ^e^	1.02 ± 0.11 ^d^	0.70 ± 0.02 ^d e^	9.59 ± 0.23
	72	15.34 ± 0.58 ^e^	1.62 ± 0.07 ^e^	0.75 ± 0.01 ^e^	9.68 ± 0.14
**Commercial Storage Stage**	**Time (days)**	**3-DG (mg·L^−1^)**	**MGO (mg·L^−1^)**	**HMF (mg·L^−1^)**	**Acrylamide (μg·L^−1^)**
	0	8.77 ± 0.52 ^a^	0.94 ± 0.04 ^a^	0.20 ± 0.02 ^NS^	-
	5	10.31 ± 1.12 ^b^	1.16 ± 0.11 ^b^	0.24 ± 0.04	-
	10	7.61 ± 0.47 ^c^	1.34 ± 0.08 ^b^	0.18 ± 0.04	-
	15	7.75 ± 0.43 ^c^	1.32 ± 0.05 ^b^	0.22 ± 0.03	-
	21	7.41 ± 0.37 ^c^	1.38 ± 0.06 ^b^	0.21 ± 0.02	-

3-DG, deoxyglucosone; MGO, methylglyoxal; HMF, 5-(hydroxymethyl)-2-furfural. Data are presented as mean values ± SDs from three independent experiments. Different lowercase letters in a column of each stage indicate significant differences (*p* < 0.05). ^NS^, no significant difference. “-”, not detected.

**Table 2:** Comparisons between flavour component levels during browning (180 min) and fermentation (72 h) of brown fermented milk (BFM).

Number	Flavour Component	Browning Stage (180 min) %	Fermentation Stage (72 h) %	RI	Rt (min)
	Alcohols				
1	1-Hexanol	2.42 ± 0.12	0.23 ± 0.09	867	14.63
2	1-Octanol	0.62 ± 0.19	0.18 ± 0.08	984	20.24
3	Trans-2-dodecen-1-ol	2.41 ± 0.21	-	1307	9.10
4	2-Furanmethanol	4.70 ± 0.34	4.54 ± 0.15	862	23.86
5	1-Dodecanol	0.64 ± 0.07	-	1277	19.03
6	Tridecyl alcohol	2.01 ± 0.04	2.14 ± 0.18	1140	19.86
7	3,7-Dimethyl-2,6-octadiex-1-ol	0.23 ± 0.06	-	987	10.82
8	(2,5-Dimethyl-3-furyl) methanol	0.20 ± 0.02	0.13 ± 0.02	1735	7.38
9	1-Nonanol	-	0.82 ± 0.17	1171	16.85
10	Cyclopropyl carbinol	-	0.64 ± 0.09	859	11.48
11	1-Undecylalcohol	-	0.20 ± 0.05	1329	17.36
12	Decylalcohol	-	0.69 ± 0.06	1259	18.94
13	5-Decen-1-ol	-	0.31 ± 0.02	1139	19.34
14	1-Cyclododecanol	-	0.20 ± 0.03	1027	21.29
15	1-Hexadecanol	-	0.97 ± 0.12	1530	20.54
	Carboxylic acids				
16	Phthalic acid	16.6 ± 0.68	1.88 ± 0.24	2719	29.47
17	Hexanoic acid	4.17 ± 0.44	5.28 ± 0.05	1946	28.99
18	Butanoic acid	23.46 ± 1.14	27.19 ± 0.32	801	23.10
19	Octanoic acid	-	5.73 ± 0.85	2189	34.23
20	Acetic acid	-	22.17 ± 1.06	1463	15.79
21	Benzoic acid	-	4.21 ± 0.79	2589	18.93
22	Propionic acid	0.72 ± 0.15	-	1595	16.48
23	n-Decanoic acid	-	1.76 ± 0.24	1349	40.12.
	Aldehydes				
24	Tridecanal	5.52 ± 0.82	1.18 ± 0.13	1103	44.12
25	Acetaldehyde	4.12 ± 0.24	3.13 ± 0.05	707	1.42
26	Octanal	1.60 ± 0.28	-	1292	28.34
27	Furfural	1.65 ± 0.08	1.05 ± 0.18	834	17.83
28	1-Nonanal	3.22 ± 0.39	-	1091	35.30
29	Benzaldehyde	2.84 ± 0.33	-	964	14.92
	Ketones				
30	Acetone	-	2.31 ± 0.12	812	6.91
31	5-Undecanone	-	0.21 ± 0.12	1597	43.66
32	2-Tridecanone	0.99 ± 0.19	0.31 ± 0.05	1423	38.23
33	Nonanone	0.98 ± 0.13	0.13 ± 0.01	1389	15.45
34	2-Pentadecanone	2.75 ± 0.37	0.24 ± 0.04	2022	41.23
35	2H-Pyran-2-one	2.71 ± 0.15	2.12 ± 0.02	1306	32.49
36	Nonalactone	0.82 ± 0.19	0.3 ± 0.08	1507	37.12
37	6-Methyl-5-hepten-2-one	0.8 ± 0.22	-	845	22.34
	Others				
38	2-[2-(2-methoxyethoxy)ethoxy]-2-methylpropane	-	0.34 ± 0.09	1458	7.96
39	4-Dodecanolide	0.63 ± 0.15	-	1302	15.68
40	Dibutylphthalate	2.82 ± 0.42	0.26 ± 0.06	2107	36.59
41	Dodecane	1.05 ± 0.30	0.11 ± 0.02	1200	14.58

All compounds were identified using MS and RI; MS: identification based on the NIST MS library; RI: identification based on retention index. Data are presented as mean values ± SDs from three independent experiments. Percentage of flavour component is based on the area normalisation. “-”, not detected.

**Table 3:** Changes in the colour parameters (a*, b*, L*, *ΔE* and BI) during the manufacturing and storage of brown fermented milk (BFM).

**Browning Stage**	**Time (min)**	a*	b*	L*	ΔE*	**BI**
	60	−2.16 ± 0.15 ^a^	10.32 ± 0.08 ^a^	88.79 ± 0.2 ^a^	-	59.01 ± 0.31 ^a^
	90	0.15 ± 0.21 ^b^	13.59 ± 0.18 ^b^	85.96 ± 0.11 ^b^	4.90 ± 0.28 ^a^	61.01 ± 0.14 ^b^
	120	0.35 ± 0.26 ^b^	13.74 ± 0.21 ^b^	85.66 ± 0.13 ^b^	5.27 ± 0.19 ^a^	61.19 ± 0.46 ^b^
	150	2.39 ± 0.09 ^c^	15.1 ± 0.15 ^c^	83.37 ± 0.03 ^c^	8.53 ± 0.37 ^b^	62.51 ± 0.26 ^c^
	180	3.16 ± 0.18 ^d^	15.8 ± 0.04 ^d^	82.13 ± 0.13 ^d^	10.13 ± 0.35 ^c^	63.15 ± 0.28 ^d^
**Fermentation Stage**	**Time (h)**	a*	b*	L*	ΔE*	**BI**
	12	4.19 ± 0.06 ^NS^	16.65 ± 0.05 ^NS^	80.41 ± 0.04 ^NS^	-	64.01 ± 0.49 ^NS^
	24	4.18 ± 0.11	16.63 ± 0.09	80.35 ± 0.10	0.06 ± 0.03 ^NS^	64.01 ± 0.35
	36	4.24 ± 0.08	16.67 ± 0.11	80.38 ± 0.07	0.06 ± 0.02	64.04 ± 0.58
	48	4.21 ± 0.09	16.71 ± 0.14	80.33 ± 0.15	0.10 ± 0.05	64.06 ± 0.26
	60	4.36 ± 0.20	16.53 ± 0.23	80.24 ± 0.18	0.24 ± 0.15	64.01 ± 0.32
	72	4.27 ± 0.18	16.78 ± 0.06	80.23 ± 0.09	0.24 ± 0.14	64.12 ± 0.36
**Commercial Storage Stage**	**Time (days)**	a*	b*	L*	ΔE*	**BI**
	0	2.31 ± 0.16 ^NS^	14.52 ± 0.13 ^NS^	85.36 ± 0.34 ^NS^	-	62.07 ± 0.40^NS^
	5	2.37 ± 0.21	14.57 ± 0.19	85.25 ± 0.29	2.07 ± 0.23 ^NS^	62.12 ± 0.02
	10	2.29 ± 0.13	14.38 ± 0.16	85.43 ± 0.33	1.95 ± 0.18	62.00 ± 0.38
	15	2.26 ± 0.08	14.46 ± 0.22	85.39 ± 0.36	2.18 ± 0.34	62.03 ± 0.28
	21	2.35 ± 0.11	14.51 ± 0.10	85.35 ± 0.45	2.12 ± 0.29	62.08 ± 0.13

L*, lightness; a*, greenness/redness; b*, blueness/yellowness; ΔE*, total colour difference; BI, browning index. Data represent mean values ± SDs, obtained from six independent experiments. Different lowercase letters in a column of each stage indicate significant differences (*p* < 0.05). ^NS^, no significant difference. “-”, not detected.

## References

[1] Zhi-Yuan X.U., Yan W.U., Guo B.H., Zhou L.H., Wang Y.Y., Lian-Zhong A.I. (2010). Research and preparation of a brown milk drink with probiotic. Sci. Technol. Food Industry.

[2] Asikin Y., Kamiya A., Mizu M., Takara K., Tamaki H., Wada K. (2014). Changes in the physicochemical characteristics, including flavour components and Maillard reaction products, of non-centrifugal cane brown sugar during storage. Food Chem..

[3] Silva H.L.A., Balthazar C.F., Esmerino E.A., Neto R.P.C., Rocha R.S., Moraes J., Cavalcanti R.N., Franco R.M., Tavares M.I.B., Santos J.S. (2018). Partial substitution of NaCl by KCl and addition of flavor enhancers on probiotic Prato cheese: A study covering manufacturing, ripening and storage time. Food Chem..

[4] Dong H., Rowland I., Thomas L.V., Yaqoob P. (2013). Immunomodulatory effects of a probiotic drink containing Lactobacillus casei Shirota in healthy older volunteers. Eur. J. Nutr..

[5] Ford A.C., Quigley E.M.M., Lacy B.E., Lembo A.J., Saito Y.A., Schiller L.R., Soffer E.E., Spiegel B.M.R., Moayyedi P. (2014). Efficacy of Prebiotics, Probiotics, and Synbiotics in Irritable Bowel Syndrome and Chronic Idiopathic Constipation: Systematic Review and Meta-analysis. Am. J. Gastroentero..

[6] Balthazar C.F., Silva H.L.A., Esmerino E.A., Rocha R.S., Moraes J., Carmo M.A.V., Azevedo L., Camps I.K.D., Abud Y., Sant’Anna C. (2018). The addition of inulin and Lactobacillus casei 01 in sheep milk ice cream. Food Chem..

[7] Martins S., Jongen W., van Boekel M. (2000). A review of Maillard reaction in food and implications to kinetic modelling. Trends Food Sci. Technol..

[8] Degen J., Hellwig M., Henle T. (2012). 1,2-Dicarbonyl Compounds in Commonly Consumed Foods. J. Agric. Food Chem..

[9] Rakete S., Klaus A., Glomb M.A. (2014). Investigations on the Maillard Reaction of Dextrins during Aging of Pilsner Type Beer. J. Agric. Food Chem..

[10] Rannou C., Laroque D., Renault E., Prost C., Sérot T. (2016). Mitigation strategies of acrylamide, furans, heterocyclic amines and browning during the Maillard reaction in foods. Food Res. Int..

[11] Katsaiti T., Granby K. (2016). Mitigation of the processing contaminant acrylamide in bread by reducing asparagine in the bread dough. Food Addit. Contam. Part A..

[12] Mottram D.S., Wedzicha B.L., Dodson A.T. (2002). Food chemistry: Acrylamide is formed in the Maillard reaction. Nature.

[13] Dan T., Wang D., Jin R.L., Zhang H.P., Zhou T.T., Sun T.S. (2017). Characterization of volatile compounds in fermented milk using solid-phase microextraction methods coupled with gas chromatography-mass spectrometry. J. Dairy Sci..

[14] Abordo E.A., Minhas H.S., Thornalley P.J. (1999). Accumulation of α-oxoaldehydes during oxidative stress: A role in cytotoxicity. Biochem. Pharmacol..

[15] Cornelis T., Eloot S., Vanholder R., Glorieux G., van der Sande F.M., Scheijen J.L., Leunissen K.M., Kooman J.P., Schalkwijk C.G. (2015). Protein-bound uraemic toxins, dicarbonyl stress and advanced glycation end products in conventional and extended haemodialysis and haemodiafiltration. Nephrol. Dial. Transplant..

[16] Navarro M., Atzenbeck L., Pischetsrieder M., Morales F.J. (2016). Investigations on the Reaction of C3 and C6 α-Dicarbonyl Compounds with Hydroxytyrosol and Related Compounds under Competitive Conditions. J. Agric. Food Chem..

[17] Zhang Z., Zou Y., Wu T., Huang C., Pei K., Zhang G., Lin X., Bai W., Ou S. (2016). Chlorogenic acid increased 5-hydroxymethylfurfural formation when heating fructose alone or with aspartic acid at two pH levels. Food Chem..

[18] Nguyen H.T., van der Fels-Klerx H.J., van Boekel M. (2017). Acrylamide and 5-hydroxymethylfurfural formation during biscuit baking. Part II: Effect of the ratio of reducing sugars and asparagine. Food Chem..

[19] Capuano E., Fogliano V. (2011). Acrylamide and 5-hydroxymethylfurfural (HMF): A review on metabolism, toxicity, occurrence in food and mitigation strategies. LWT Food Sci. Technol..

[20] Akıllıoglu H.G., Mogol B.A., Gökmen V. (2011). Degradation of 5-hydroxymethylfurfural during yeast fermentation. Food Addit. Contam. Part A..

[21] Baardseth P., Blom H., Skrede G., Mydland L.T., Skrede A., Slinde E. (2006). Lactic acid fermentation reduces acrylamide formation and other Maillard reactions in french fries. J. Food Sci..

[22] Reps A., Hammond E.G., Glatz B.A. (1987). Carbonyl Compounds Produced by the Growth of Lactobacillus bulgaricus1. J Dairy Sci..

[23] Bartkiene E., Jakobsone I., Juodeikiene G., Vidmantiene D., Pugajeva I., Bartkevics V. (2013). Study on the reduction of acrylamide in mixed rye bread by fermentation with bacteriocin-like inhibitory substances producing lactic acid bacteria in combination with Aspergillus niger glucoamylase. Food Control..

[24] Wang S., Yu J., Xin Q., Wang S., Copeland L. (2017). Effects of starch damage and yeast fermentation on acrylamide formation in bread. Food Control..

[25] Arena E., Ballistreri G., Tomaselli F., Fallico B. (2011). Survey of 1,2-Dicarbonyl Compounds in Commercial Honey of Different Floral Origin. J. Food Sci..

[26] Arribas-Lorenzo G., Morales F.J. (2010). Analysis, Distribution, and Dietary Exposure of Glyoxal and Methylglyoxal in Cookies and Their Relationship with Other Heat-Induced Contaminants. J. Agric. Food Chem..

[27] Hellwig M., Degen J., Henle T. (2010). 3-deoxygalactosone, a “new” 1,2-dicarbonyl compound in milk products. J. Agric. Food Chem..

[28] Mesías M., Morales F. (2017). Effect of Different Flours on the Formation of Hydroxymethylfurfural, Furfural, and Dicarbonyl Compounds in Heated Glucose/Flour Systems. Foods.

[29] Van Der Fels-Klerx H.J., Capuano E., Nguyen H.T., Ataç Mogol B., Kocadağlı T., Göncüoğlu T.N., Hamzalıoğlu A., Van Boekel M., Gökmen V. (2014). Acrylamide and 5-hydroxymethylfurfural formation during baking of biscuits: NaCl and temperature–time profile effects and kinetics. Food Res. Int..

[30] Mogol B.A., Gokmen V. (2016). Effect of chitosan on the formation of acrylamide and hydroxymethylfurfural in model, biscuit and crust systems. Food Funct..

[31] Stadler R.H., Blank I., Varga N., Robert F., Hau J., Guy P.A., Robert M.-C., Riediker S. (2002). Food chemistry: Acrylamide from Maillard reaction products. Nature.

[32] Arvanitoyannis I.S., Dionisopoulou N. (2014). Acrylamide: Formation, Occurrence in Food Products, Detection Methods, and Legislation. Crit. Rev. Food Sci. Nutr..

[33] Anese M., Manzocco L., Calligaris S., Nicoli M.C. (2013). Industrially Applicable Strategies for Mitigating Acrylamide, Furan, and 5-Hydroxymethylfurfural in Food. J. Agric. Food Chem..

[34] Pfeifer Y.V., Haase P.T., Kroh L.W. (2013). Reactivity of Thermally Treated α-Dicarbonyl Compounds. J. Agric. Food Chem..

[35] Gobert J., Glomb M.A. (2009). Degradation of Glucose: Reinvestigation of Reactive α-Dicarbonyl Compounds. J. Agric. Food Chem..

[36] Chen S.-L., Jin S.-Y., Chen C.-S. (2005). Relative reactivities of glucose and galactose in browning and pyruvaldehyde formation in sugar/glycine model systems. Food Chem..

[37] Albalá-Hurtado S., Veciana-Nogués M.T., Mariné-Font A., Vidal-Carou M.C. (1998). Changes in Furfural Compounds during Storage of Infant Milks. J. Agric. Food Chem..

[38] Hee C.I., Lee S., Jun H.-R., Roh H.-J., Kim Y.-S. (2010). Comparison of volatile Maillard reaction products from tagatose and other reducing sugars with amino acids. Food Sci. Biotechnol..

[39] Devi A.F., Buckow R., Singh T., Hemar Y., Kasapis S. (2015). Colour change and proteolysis of skim milk during high pressure thermal–processing. J. Food Eng..

[40] Tosun I., Sule Ustun N. (2003). Nonenzymic browning during storage of white hard grape pekmez (Zile pekmezi). Food Chem..

[41] Lo C.-Y., Li S., Wang Y., Tan D., Pan M.-H., Sang S., Ho C.-T. (2008). Reactive dicarbonyl compounds and 5-(hydroxymethyl)-2-furfural in carbonated beverages containing high fructose corn syrup. Food Chem..

[42] Kocadağli T., Gökmen V. (2016). Multiresponse kinetic modelling of Maillard reaction and caramelisation in a heated glucose/wheat flour system. Food Chem..

[43] Rufian-Henares J.A., Arribas-Lorenzo G., Morales F.J. (2007). Acrylamide content of selected Spanish foods: Survey of biscuits and bread derivatives. Food Addit. Contam. Part A..

[44] Roldan M., Loebner J., Degen J., Henle T., Antequera T., Ruiz-Carrascal J. (2015). Advanced glycation end products, physico-chemical and sensory characteristics of cooked lamb loins affected by cooking method and addition of flavour precursors. Food Chem..

[45] Contador R., Delgado F.J., García-Parra J., Garrido M., Ramírez R. (2015). Volatile profile of breast milk subjected to high-pressure processing or thermal treatment. Food Chem..

